# Integrating Genotyping and Left Atrial Strain Analysis Enhances Risk Stratification in Hypertrophic Cardiomyopathy

**DOI:** 10.1016/j.jacadv.2026.102863

**Published:** 2026-06-22

**Authors:** Takashi Hiruma, Koki Nakanishi, Shunsuke Inoue, Seitaro Nomura, Yuriko Yoshida, Zhehao Dai, Ryo Abe, Takanobu Yamada, Kanna Fujita, Manami Katoh, Toshiyuki Ko, Masamichi Ito, Junichi Ishida, Eisuke Amiya, Masaru Hatano, Norifumi Takeda, Hiroyuki Morita, Norihiko Takeda, Issei Komuro

**Affiliations:** a Department of Cardiovascular Medicine, Graduate School of Medicine, The University of Tokyo, Tokyo, Japan; b Department of Frontier Cardiovascular Science, The University of Tokyo Graduate School of Medicine, Tokyo, Japan; c Department of Pathophysiology of Heart Failure and Therapeutics, National Cerebral & Cardiovascular Center, Osaka, Japan; d Advanced Medical Center for Heart Failure, The University of Tokyo Hospital, Tokyo, Japan; e The Institute of Medical Science, The University of Tokyo, Tokyo, Japan; f International University of Health and Welfare, Tokyo, Japan

**Keywords:** hypertrophic cardiomyopathy, genetics, left atrium, strain analysis, systolic dysfunction

## Abstract

**Background:**

Patients with hypertrophic cardiomyopathy (HCM) who progress to left ventricular systolic dysfunction (LVSD) are at an increased risk of lethal arrhythmias and end-stage heart failure. Early identification of this high-risk subpopulation is crucial; however, current risk stratification remains insufficient.

**Objectives:**

The objective of the study was to investigate the association of myocardial strain parameters with progression to LVSD in genotyped HCM patients.

**Methods:**

Sarcomere positive was defined as harboring pathogenic or likely pathogenic variants in sarcomere-encoding genes. Speckle-tracking echocardiography was used to measure left ventricular global longitudinal strain (LVGLS) and left atrial reservoir strain (LARS). The primary endpoint was progression to LVSD (ejection fraction <50%) and the secondary endpoint was a composite of progression to LVSD, heart-failure hospitalization, and all-cause death.

**Results:**

A total of 101 patients (23.8% sarcomere positive) were included and followed up for 5.0 (IQR: 2.4-8.7) years. No significant association was observed between genotype and strain parameters. Sarcomere positive (subdistribution HR [sHR]: 5.07; 95% CI: 1.25 to 20.6; *P* = 0.023) and lower LARS (sHR: 1.10 per 1% decrease; 95% CI: 1.02-1.18; *P* = 0.015), but not LVGLS, were significantly associated with the primary endpoint. Sarcomere-positive patients with reduced LARS faced markedly elevated incidence for the primary endpoint (sHR: 8.31; 95% CI: 2.25-30.7; *P* = 0.001). Consistent findings were observed in terms of the secondary endpoint (HR: 4.76; 95% CI: 1.59-14.2; *P* = 0.005).

**Conclusions:**

LARS was associated with progression to LVSD in HCM, potentially reflecting the preclinical stage of LVSD, whereas LVGLS was not. In this exploratory study, integrating left atrial strain analysis with genotyping may have potential value in risk stratification, with implication for early detection of vulnerable individuals.

Hypertrophic cardiomyopathy (HCM) is a common inherited myocardial disease, with an estimated prevalence of 1 in 200 to 500 individuals in the general population.^1^ Patients with HCM exhibit various clinical phenotypes, such as intraventricular obstruction, lethal arrhythmias, and heart failure. Notably, about 10% of them progress to left ventricular (LV) systolic dysfunction (LVSD), an advanced phenotype requiring multidisciplinary therapeutics accompanied by a high social burden.^1^, ^2^, ^3^, ^4^ This underscores the importance of early identification of individuals at risk of progressing to LVSD, as well as the development of effective preventive strategies. Although germline variants in genes encoding myocardial sarcomeric proteins are strong predictors for this, patients with variants in the same gene, or even the same variants do not follow completely same clinical courses, suggesting the need for novel predictive markers capable of capturing preclinical adverse cardiac remodeling.^5^, ^6^, ^7^ In this regard, the presence of late gadolinium enhancement and elevated cardiac troponin T levels have been implicated as predictive factors of future LVSD.^4^^,^^8^

Echocardiography is incorporated into routine workups for HCM as a noninvasive imaging modality. Over the past few decades, myocardial strain analysis using speckle-tracking echocardiography has emerged as a pivotal modality for detecting subclinical myocardial dysfunction before overt deterioration of LV ejection fraction (LVEF).^9^^,^^10^ In addition, myocardial strain analysis using cardiac magnetic resonance imaging also provides mechanistic insights into disease progression in HCM.^11^^,^^12^ Given that echocardiography-derived myocardial strain analysis is more accessible and readily available in routine clinical practice, it may serve as a practical screening modality. Prior studies have demonstrated the utility of echocardiography-derived myocardial strain parameters for prognostic stratification in HCM;^13^, ^14^, ^15^, ^16^, ^17^ however, their association with progression to LVSD remains poorly understood. Furthermore, the relationship between genetic predispositions and strain parameters has yet to be elucidated. In this study, we conducted myocardial strain analysis in a genotyped HCM cohort to: 1) explore the relationship between genotype and strain parameters; and 2) evaluate the potential utility of integrating genotyping and strain parameters for risk stratification in relation to LVSD and adverse clinical outcomes.

## Methods

### Study population and study design

This retrospective study included 151 consecutive genotyped patients who were clinically diagnosed with familial or sporadic HCM and underwent transthoracic echocardiography between April 2012 and August 2024 at the University of Tokyo Hospital, where the highest number of heart transplantations has been performed in Japan. Patients with a history of surgical myectomy, alcohol septal ablation, LV assist device implantation, or heart transplantation, as well as those with storage or systemic diseases (e.g. Danon disease, Fabry disease, *PRKAG2* syndrome desminopathy, and cardiac amyloidosis), which are phenocopies of HCM, or with suboptimal image quality for conventional or strain analysis of both the left atrium (LA) and LV were excluded from the analysis. In addition, patients who had already progressed to LVSD (LVEF <50%) at the initial echocardiography were also excluded.

Clinical data, including medical history, medications, cardiac electronic implanted devices, and blood tests, were collected at the time of the initial echocardiography. All data were obtained by physicians and assistant staff who were blinded to the genetic results, and clinical and survival status were ascertained through May 31, 2025. A family history of HCM was defined as the presence of at least 1 affected relative with clinical evidence of HCM. A family history of sudden cardiac death (SCD) was recorded if any family member had experienced an unexpected death, regardless of age or presumed cause.

This study was approved by the Ethics Committee of the University of Tokyo Hospital (approval number: G2249) and conducted following the principles of the Declaration of Helsinki. Written informed consent was obtained from all the participants. All supporting data are available from the corresponding author on reasonable request.

### HCM diagnosis, classification, and management

The diagnosis and classification of HCM were based on 2-dimensional echocardiography findings according to current guidelines.^18^^,^^19^ HCM was defined as unexplained LV hypertrophy with a maximal end-diastolic LV wall thickness of ≥15 mm at any LV segment, or ≥13 mm in individuals with a known family history of HCM. LV obstruction was defined as an elevated intra-LV gradient ≥30 mm Hg, observed either at rest or during provocable testing. LVSD was defined as a reduced LVEF <50%. Septal morphology was categorized into reverse curve, sigmoid, apical, and neutral phenotypes.^20^ All patients were managed according to contemporary guideline-directed medical therapy.^18^^,^^19^ As cardiac myosin inhibitors were not approved in Japan during the study period, no patients received cardiac myosin inhibitors in this cohort.

### Conventional echocardiography

Conventional echocardiography was performed using commercially available ultrasound systems with the earliest available data from April 2012 to August 2024 analyzed for each patient. All echocardiographic examinations were conducted by experienced operators. The linear dimensions of cardiac chambers were measured according to the recommendations of the American Society of Echocardiography.^21^ LVEF and LA volume were evaluated using the Simpson’s method. LA volume was indexed to body surface area (LA volume index [LAVI]). LV diastolic function was assessed according to the guideline published in 2016.^22^ Briefly, pulsed-wave Doppler examination of mitral inflow was performed to measure early (E) and late (A) peak velocities from the apical 4-chamber view. Mitral annular velocities were determined by tissue Doppler imaging, and the peak early diastolic velocities of the septal and lateral sides were measured. Each E/e′ ratio was then calculated as an estimate of LV filling pressure.

### Myocardial strain analysis with speckle-tracking echocardiography

Speckle-tracking echocardiography was performed by experienced cardiologists using vendor-independent commercial software (2-dimensional Cardiac Performance Analysis; Tomtec Imaging System), as previously described.^10^ Endocardial borders of each chamber were semiautomatically traced and tracked throughout the cardiac cycle, with manual adjustments made in cases of suboptimal tracking. LV global longitudinal strain (LVGLS) was calculated by averaging the negative peak of longitudinal strain from all 3 apical views, including the 4-chamber, 2-chamber, and long-axis views.^23^ LA reservoir strain (LARS) was obtained by averaging peak values of LA segmental strains from apical 4-chamber view.^24^^,^^25^ LA pump and conduit strains were also assessed in patients in sinus rhythm at the time of the initial echocardiography. According to the definition of strain parameters, negative values for LVGLS indicate myocardial shortening, meaning that higher absolute values reflect better LV systolic function. In contrast, LARS is expressed as a positive value, with higher values indicating better LA reservoir function. Interobserver variabilities for LVGLS and LARS were analyzed in 15 randomly selected patients and assessed by 2 independent and blinded observers using Bland-Altman analysis.

### Genetic analysis and variant classification

Genetic testing was conducted as previously described.^6^ DNA was extracted from whole blood samples. Target sequencing was performed in 30 patients using a custom panel targeting exons and splice site regions of 83 genes associated with various cardiomyopathies and arrhythmias (Supplemental Table 1). Whole exome sequencing was conducted in the remaining 121 patients. The pathogenicity of the variants was evaluated and classified as pathogenic, likely pathogenic, variant of uncertain significance, benign, or likely benign, based on the consensus guidelines of the American College of Medical Genetics and Genomics and the latest recommendations from ClinGen.^26^^,^^27^ Detailed methods are provided in the Supplemental Methods.

Following definitions from previous studies and the ClinGen Hereditary Cardiovascular Disease Gene Curation Expert Panel,^28^ patients harboring pathogenic or likely pathogenic (P/LP) variants in 9 major sarcomere-encoding genes which are definitive for HCM onset were classified as “sarcomere positive.” These genes include *MYBPC3, MYH7*, *MYL2*, *MYL3*, *TNNT2*, *TNNI3*, *TNNC1*, *TPM1,* and *ACTC1*. Patients with variants of uncertain significance, likely benign or benign variants, or those without sarcomeric variants were classified as “sarcomere negative.” Moreover, patients harboring P/LP variants associated with other cardiovascular diseases (CVDs), such as dilated cardiomyopathy, arrhythmogenic cardiomyopathy and arrhythmias, were referred to as “other CVD-related variants positive.”^6^

### Follow-up and study endpoints

All patients were followed up at the University of Tokyo Hospital and the clinical evaluation and management were conducted at the discretion of the treating physician. The primary endpoint was the progression to LVSD during follow-up. The secondary endpoint was a composite of the progression to LVSD, hospitalization for heart failure, and all-cause death, with time to the first event used for analysis.

### Statistical analysis

Categorical variables were presented as numbers with percentages. Continuous variables were expressed as median with IQRs. Comparisons between groups were performed using the Mann-Whitney *U* test for continuous variables and the chi-square test or Fisher exact test, as appropriate, for categorical variables.

To assess the association of genotype and strain parameters with the primary endpoint, competing risk analyses were performed with all-cause death treated as a competing event. Subdistribution HRs (sHRs) with 95% CIs were estimated using Fine-Gray proportional subdistribution hazard models. Cumulative incidences were compared using Gray test. The proportional subdistribution hazards assumption was evaluated by testing time-varying effects using interactions between each covariate and follow-up time. For the secondary endpoint, time-to-event was analyzed using Cox proportional hazards models. The proportional hazards assumption for Cox models was evaluated using Schoenfeld residuals for each covariate. Cumulative incidence was estimated using Kaplan-Meier curves and compared with the log-rank test. In both models, only variables that satisfied the proportional (subdistribution) hazards assumption were included and only univariate analyses were conducted due to the limited number of study endpoints.

The extent of missing data was summarized in Supplemental Table 2. Missing data were handled by multiple imputation using chained equations under the assumption of missing at random. All variables listed in Tables 1 and 2 were included in the imputation model. A total of 20 imputed data sets were generated using fully conditional specification with predictive mean matching and the results across imputations were combined using Rubin rules.^29^ Multiple imputation was applied only to regression analyses, whereas descriptive statistics were based on observed data.

**Table 1:** Echocardiographic Parameters at the Initial Evaluation

	N	Sarcomere Positive (n = 24)	Sarcomere Negative (n = 77)	*P* Value
Septal morphology	101			<0.001
Reverse curve septum, %		21 (87.5)	17 (22.1)	
Sigmoidal septum, %		0 (0.0)	32 (41.6)	
Apical dominant, %		0 (0.0)	16 (20.8)	
Neutral, %		3 (12.5)	12 (15.6)	
LV parameters				
Interventricular septal wall thickness, mm	101	17 (14-22)	13 (12-16)	<0.001
Posterior wall thickness, mm	101	9 (8-11)	10 (9-13)	0.081
LV maximal wall thickness, mm	101	20 (15-23)	15 (14-18)	0.009
LV end-diastolic volume, mL	101	95.4 (78.4-130)	93.7 (67.5-119)	0.15
LV end-systolic volume, mL	101	36.0 (26.1-50.1)	30.6 (22.4-43.2)	0.16
LV ejection fraction, %	101	62 (59-68)	64 (60-69)	0.35
LV global longitudinal strain, %	87	−16.4 (−19.1/-14.7)	−15.3 (−18.2/-13.0)	0.13
E wave, cm/sec	99	54.1 (49.6-73.6)	65.1 (52.1-84.6)	0.11
Septal E/e’ ratio	97	12.2 (9.9-15.6)	15.1 (11.1-19.6)	0.053
Lateral E/e’ ratio	94	9.7 (7.4-10.6)	10.6 (7.5-14.1)	0.25
Intra-LV pressure gradient, mm Hg	101	0 (0-8.0)	13.4 (0-48)	<0.001
Intra-LV obstruction, %	101	0 (0.0)	27 (35.1)	<0.001
Apical hypertrophy, %	101	0 (0.0)	18 (23.4)	0.006
Apical aneurysm, %	101	0 (0.0)	4 (5.2)	0.57
LA parameters				
LA diameter, mm	101	40.5 (36.3-49.8)	42.0 (35.0-45.0)	0.86
LA volume index, mL/m^2^	101	48.9 (34.6-65.7)	43.9 (33.6-60.1)	0.50
LA reservoir strain, %	101	22.6 (14.2-28.3)	22.1 (17.9-26.5)	0.80
LA pump strain, %	99	7.2 (5.4-13.2)	10.3 (6.1-15.0)	0.093
LA conduit strain, %	99	11.9 (8.6-19.1)	9.9 (7.5-15.0)	0.10
Valvular heart disease				
Aortic regurgitation grade ≥2+, %	101	0 (0.0)	0 (0.0)	1.00
Mitral regurgitation grade ≥2+, %	101	3 (12.5)	10 (13.0)	1.00
Tricuspid regurgitation grade ≥2+ %	101	0 (0.0)	2 (2.6)	1.00

Values are median (IQR) or n (%).

*P* value indicates comparison between sarcomere-positive vs sarcomere-negative patients using the Mann-Whitney *U* test for continuous variables and the Fisher exact test for categorical variables.

LA = left atrium; LV = left ventricle.

**Table 2:** Demographic Data of the Patients at the Initial Evaluation

	N	Sarcomere Positive (n = 24)	Sarcomere Negative (n = 77)	*P* Value
Age at diagnosis of HCM, years	101	40 (22-51)	62 (57-70)	<0.001
Age at echocardiography, years	101	53 (32-57)	66 (58-72)	<0.001
Female, %	101	12 (50.0)	35 (45.5)	0.82
Body mass index, kg/m^2^	101	22.9 (20.8-25.1)	23.5 (21.7-26.8)	0.13
Family history of HCM, %	101	12 (50.0)	9 (11.7)	<0.001
Family history of SCD, %	101	10 (41.7)	2 (2.6)	<0.001
NYHA function class Ⅲ/IV, %	101	1 (4.2)	9 (11.7)	0.44
The HCM risk-SCD score, %	101	2.33 (1.82-4.27)	1.38 (1.01-2.44)	<0.001
Prior unexpected syncope, %	101	9 (37.5)	12 (15.6)	0.040
Prior hospitalization for heart failure, %	101	3 (12.5)	2 (2.6)	0.086
History of atrial fibrillation	101			1.00
Paroxysmal, %		4 (16.7)	9 (11.7)	
Persistent, %		1 (4.2)	4 (5.2)	
Other CVD-related variants positive, %	101	4 (16.7)	1 (1.3)	0.011
Comorbidities				
Hypertension, %	101	8 (33.3)	40 (51.9)	0.16
Diabetes, %	101	3 (12.5)	14 (18.2)	0.76
Dyslipidemia, %	101	11 (45.8)	41 (53.2)	0.64
Chronic kidney disease, %	101	3 (12.5)	11 (14.3)	1.00
Coronary artery disease, %	101	0 (0.0)	7 (9.1)	0.19
Blood test at echocardiography				
Hemoglobin, g/dL	101	14.1 (12.9-15.2)	13.8 (12.9-15.1)	0.90
Estimated GFR, mL/min/1.73 m^2^	100	72.5 (63.8-86.4)	62.0 (54.2-70.6)	0.020
C-reactive protein, mg/dL	94	0.08 (0.03-0.16)	0.06 (0.03-0.18)	0.89
B-type natriuretic peptide, pg/mL	99	293 (113-509)	125 (53.7-233)	0.008
Cardiac troponin I, pg/mL	69	25.6 (15.5-43.3)	17.7 (10.0-32.7)	0.15
Medications at echocardiography				
Beta-blocker, %	101	20 (83.3)	48 (62.3)	0.080
ACE inhibitor, ARB, or ARNI, %	101	9 (37.5)	35 (45.5)	0.64
Mineralocorticoid receptor antagonist, %	101	5 (20.8)	5 (6.5)	0.055
Diuretic agents, %	101	1 (4.2)	9 (11.7)	0.44
Nondihydropyridine CCB, %	101	3 (12.5)	7 (9.1)	0.70
Type I antiarrhythmic agents, %	101	2 (8.3)	7 (9.1)	1.00
Type III antiarrhythmic agents, %	101	4 (16.7)	1 (1.3)	0.011
Anticoagulant agents, %	101	6 (25.0)	16 (20.8)	0.78
Cardiac electronic implanted devices at echocardiography				
Permanent pacemaker, %	101	0 (0.0)	5 (6.5)	0.34
Implantable cardioverter-defibrillator, %	101	4 (16.7)	10 (13.0)	0.75

Values are median (IQR) or n (%).

*P* value indicates comparison between sarcomere-positive vs sarcomere-negative patients using the Mann-Whitney *U* test for continuous variables and the Fisher exact test for categorical variables.

ACE = angiotensin-converting enzyme; ARB = angiotensin II receptor blocker; ARNI = angiotensin receptor-neprilysin inhibitor; CCB = calcium-channel blocker; CVD = cardiovascular disease; GFR = glomerular filtration rate; HCM = hypertrophic cardiomyopathy; SCD = sudden cardiac death.

Statistical significance was defined as a 2-sided *P* value <0.05. All statistical analyses were conducted using R software (version 4.3.1; R Foundation for Statistical Computing).

## Results

### Study population

**Figure 1:**
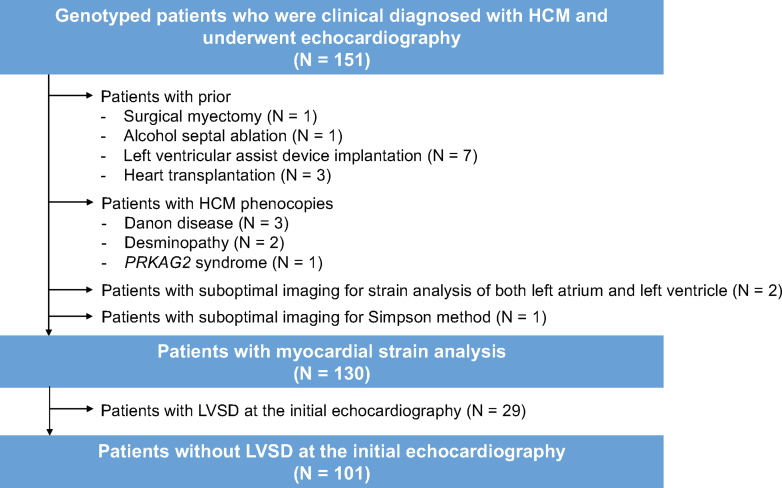
**Patients Flow of This Study** Among 151 genotyped patients with clinically diagnosed HCM, a total of 101 patients met the inclusion criteria. HCM = hypertrophic cardiomyopathy; LVSD = left ventricular systolic dysfunction.

The patient flow of this study is shown in Figure 1. Of 151 genotyped patients with clinically diagnosed HCM, 130 underwent myocardial strain analysis. At the initial evaluation, 29 patients had already progressed to LVSD (median LVEF, 36.9% [IQR: 30.7%-43.6%]). These patients had a high prevalence of sarcomere positivity (62.1%). Both LVGLS (−9.9% [IQR: −11.7% to −8.0%]) and LARS (IQR: 9.2% [6.0%-14.2%]) were substantially impaired. Detailed demographic data and echocardiographic parameters of patients with LVSD at the initial evaluation are presented in Supplemental Tables 3 and 4.

**Figure 2:**
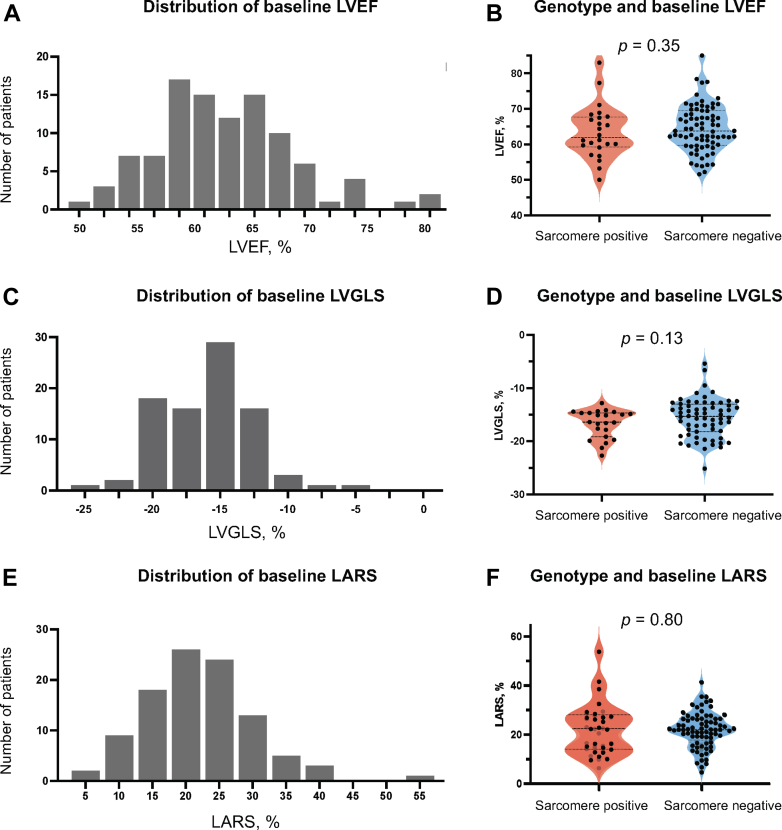
**Myocardial Strain Parameters at the Initial Echocardiography** Histograms show the distribution of (A) LVEF, (C) LVGLS, and (E) LARS. (B, D, F) Violin plots show comparisons by genotype: sarcomere positive vs negative. Violin plots display median, IQRs, and full distribution. LARS = left atrial reservoir strain; LVEF = left ventricular ejection fraction; LVGLS = left ventricular global longitudinal strain.

A total of 101 patients (age, 62 years [IQR: 55-71 years]; female, 46.5%) were included in the analysis. Eighteen patients (17.8%) had a history of atrial fibrillation (AF), of whom 13 had paroxysmal and 5 had persistent AF. At the initial echocardiography, 27 patients (26.7%) had LV obstruction and 18 (17.8%) demonstrated apical hypertrophy. The maximal LV wall thickness was 16 (IQR: 14-19) mm, predominantly located in the basal anteroseptal segment (55.4%). The median LVEF was 63.8% (IQR: 59.7%-68.5%) and its distribution is shown in Figure 2. The median LAVI was 45.2 (IQR: 34.5-61.2) mL/m^2^.

### Genotype-phenotype correlation

Genetic testing revealed that 24 patients (23.8%) were sarcomere positive. P/LP variants in sarcomere-encoding genes identified in this cohort are listed in Table 3: *MYBPC3* (n = 11), *MYH7* (n = 11), *MYL3* (n = 1), *TNNT2* (n = 1), and *TNNI3* (n = 1). One patient had double heterozygous likely pathogenic variants in *MYH7* and *MYL3*. The details of clinical characteristics and echocardiographic data stratified by genotype are presented in Tables 1 and 2, respectively. Sarcomere-positive patients were younger, had a higher prevalence of family history of HCM and SCD, had higher HCM Risk-SCD scores, higher estimated glomerular filtration rate, higher B-type natriuretic peptide levels, and were more likely to be prescribed type III antiarrhythmic agents. Four (16.7%) had coexisting other CVD-related variants. Sarcomere-positive patients had a higher prevalence of reverse curve septum and greater LV wall thickness and a lower prevalence of sigmoidal septal morphology, LV obstruction and apical hypertrophy compared with genotype-negative patients. LV volume, LVEF, E/e’ ratio, and LAVI were similar between the groups.

**Table 3:** Pathogenic or Likely Pathogenic Variants in Sarcomere-Encoding Genes Identified in This Study

Gene	Coding DNA	Protein	Variant Type	Zygosity	ACMG Criteria	ACMG Classification	Number of Patients
*MYBPC3*	c.25+1G > T	-	Splicing	Hetero	PVS1, PS1_Supporting, PM2_supporting	P	1
*MYBPC3*	c.1042_1043insCGGCA	p.M348Tfs∗4	Frameshift insertion	Hetero	PVS1, PS4_Supporting, PM2_Supporting	P	1
*MYBPC3*	c.1224-2A > G	-	Splicing	Hetero	PVS1, PS3_Supporting, PS4_Supporting, PM2_Supporting	P	1
*MYBPC3*	c.1484G > A	p.R495Q	Missense	Hetero	PS3, PS4, PM5, PM2_Supporting, PP1	P	1
*MYBPC3*	c.1777delT	p.S593Pfs∗9	Frameshift deletion	Hetero	PVS1, PS3_Moderate, PS4, PM2_Supporting, PP1_Strong	P	3
*MYBPC3*	c.1790+1G > T	-	Splicing	Hetero	PVS1, PS3, PS4_Supporting, PM2_Supporting	P	1
*MYBPC3*	c.3412delC	p.R1138Afs∗51	Frameshift deletion	Hetero	PVS1, PS4_Supporting, PM2_Supporting	P	3
*MYH7*	c.746G > A	p.R249Q	Missense	Hetero	PS2, PS3_Supporting, PS4, PM1, PM2_Supporting, PP1_Strong, PP3_Moderate	P	1
*MYH7*	c.1357C > T	p.R453C	Missense	Hetero	PS3_Supporting, PS4, PM2_Supporting, PM5, PM6_Supporting, PP1_Strong, PP3_Moderate	P	1
*MYH7*	c.1744T > C	p.Y582H	Missense	Hetero	PS3_Moderate, PS4_Supporting, PM1, PM2_Supporting, PP3_Moderate	LP	2
*MYH7*	c.1816C > A	p.V606M	Missense	Hetero	PS3_Moderate, PS4_Strong, PM2_Supporting, PM5, PP1_Strong, PP3	P	1
*MYH7*	c.2222G > T	p.G741V	Missense	Hetero	PS4_Moderate, PM2_Supporting, PM5, PP3	LP	1
*MYH7*	c.2608C > T	p.R870C	Missense	Hetero	PS4, PM5, PP3_Moderate	LP	1
*MYH7*∗	c.2653A > G	p.N885D	Missense	Hetero	PS4_Supporting, PM1, PM2_Supporting, PP3_Moderate	LP	1
*MYH7*	c.2803G > A	p.E935K	Missense	Hetero	PS4_Moderate, PM1, PM2_Supporting, PP3	LP	1
*MYH7*	c.3158G > A	p.R1053Q	Missense	Hetero	PS4, PM1, PM2_Supporting, PP1_Strong, PP3_Moderate	P	1
*MYH7*	c.5279C > G	p.T1760R	Missense	Hetero	PS4_Supporting, PM2_Supporting, PM5, PP3_Moderate	LP	1
*MYL3*∗	c.466G > A	p.V156M	Missense	Hetero	PS4_Moderate, PM2_Supporting, PM5, PP3_Moderate	LP	1
*TNNI3*	c.557C > A	p.R186Q	Missense	Hetero	PS4, PM1, PM2_Supporting, PP1_Strong	P	1
*TNNT2*	c.236T > C	p.I79T	Missense	Hetero	PS4_Supporting, PM1, PM2_Supporting, PP3_Moderate	LP	1

ACMG = American College of Medical Genetics and Genomics; LP = likely pathogenic; P = pathogenic.

∗ One patient had double heterozygous variants in *MYH7* and *MYL3*.

Distribution of myocardial strain parameters are shown in Figure 2. The median values of LVGLS, LARS, LA pump strain, and LA conduit strain were −15.4% (IQR: −18.2% to −13.8%), 22.2% (IQR: 16.4%-26.8%), 9.4% (IQR: 5.5%-14.2%), and 9.9% (IQR: 7.8-15.5), respectively. There were no significant differences in LVGLS, LARS, LA pump strain, and LA conduit strain between sarcomere-positive and genotype-negative patients. Bland-Altman analysis showed that the agreements between interobserver measurements were −0.5% ± 2.1% for LVGLS and 0.9% ± 5.6% for LARS (mean ±1.96 SD, respectively).

### Patients who progressed to LVSD

During the median follow-up period of 5.0 (IQR: 2.4-8.7) years, 8 patients (7.9%; age, 54 [IQR: 41-70] years; female, 50.0%; LVEF 59.1% [IQR: 55.9%-66.6%]) had progressed to LVSD. Of these, 5 (62.5%) were sarcomere positive: *MYBPC3* (n = 2), *MYH7* (n = 1), *TNNT2* (n = 1), and *TNNI3* (n = 1). No patient had LV obstruction. Beta-blocker was prescribed in 7 (87.5%) patients. The median interval from the initial echocardiography to LVSD was 2.4 (IQR: 1.2-3.7) years. Detailed information of these 8 patients is listed in Supplemental Table 5.

### Factors associated with the progression to LVSD

Competing risk regression analyses based on multiply imputed data sets were performed to assess factors associated with the progression to LVSD, treating all-cause death as a competing event (Table 4). A total of 3 deaths occurred as competing events for the primary endpoint. Variables that met the proportional subdistribution hazards assumption were included in the Fine-Gray model (Supplemental Table 6). In this model, sarcomere positive (sHR: 5.07; 95% CI: 1.25-20.6; *P* = 0.023) and LARS (sHR: 1.10 per 1% decrease; 95% CI, 1.02-1.18; *P* = 0.015) were significantly associated with the progression to LVSD. No patient with LV obstruction had progressed to LVSD during follow-up. Neither LA pump strain nor conduit strain was associated with the primary endpoint.

**Table 4:** Fine-Gray Proportional Subdistribution Hazard Model for Evaluating Association With the Progression to LVSD

	sHR (95% CI)	*P* Value
Age at echocardiography, per 10-year increase	0.87 (0.64-1.19)	0.38
Body mass index, per 1 kg/m^2^ increase	0.92 (0.81-1.03)	0.15
Sarcomere positive	5.07 (1.25-20.6)	0.023
LV maximal wall thickness, per 1 mm increase	0.87 (0.65-1.17)	0.36
LV end-diastolic volume, per 10 mL increase	1.01 (0.90-1.10)	0.90
LV ejection fraction, per 1% decrease	1.12 (0.99-1.26)	0.077
LV global longitudinal strain, per 1% increase	1.01 (0.88-1.15)	0.91
Septal E/e’ ratio, per 1 increase	0.94 (0.83-1.07)	0.34
LA volume index, per 10 mL/m^2^ increase	1.12 (0.99-1.26)	0.47
LA reservoir strain, per 1% decrease	1.10 (1.02-1.18)	0.015
LA pump strain, per 1% decrease	1.09 (0.94-1.26)	0.28
LA conduit strain, per 1% decrease	1.14 (0.97-1.34)	0.12
Follow-up period, per 1 y increase	1.11 (0.83-1.50)	0.47

LVSD = left ventricular systolic dysfunction; sHR = subdistribution HR; other abbreviations as in Table 3.

**Figure 3:**
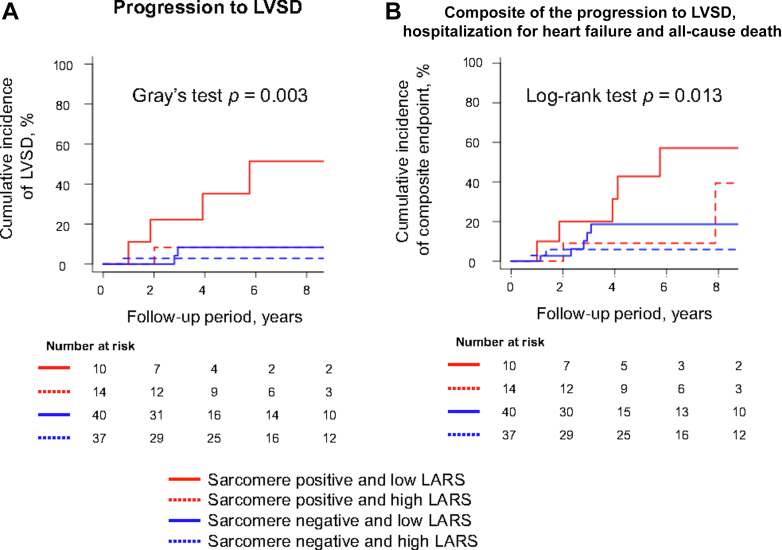
**Event Curves Stratified by Genotype and Left Atrial Reservoir Strain** Cumulative incidence of the primary endpoint (progression to LVSD) considering the competing risk of death (A, Gray test) and Kaplan-Meier curves for the secondary endpoint (composite of the progression to LVSD, hospitalization for heart failure and all-cause death) (B, Log-rank test) stratified by genotype (sarcomere positive vs negative) and LARS (above or below the median value of 22.2%). Abbreviations as in Figures 1 and 2.

As shown in Figure 3, when stratified by genotype (sarcomere positive vs negative) and LARS (high vs low divided by the median value of 22.2%), the cumulative incidence of LVSD differed significantly among the 4 groups (Gray test, *P* = 0.003). Sarcomere-positive patients with low LARS constituted a high-risk population compared to the others (5-year estimated event incidence, 31.4% [95% CI: 0.0%-63.2%] vs 5.7% [95% CI: 0.2%-11.1%]; sHR, 8.31; 95% CI: 2.25-30.7; *P* = 0.001).

### Impact of LARS on mortality and heart failure events

Composite of the progression to LVSD, heart failure–related events and all-cause death were observed in 14 patients (13.9%) including 7 hospitalizations for heart failure, 2 heart failure deaths, and 1 SCD. Variables that met the proportional hazards assumption were included in the Cox proportional analysis (Supplemental Table 7). In this model, sarcomere positive (HR: 3.10; 95% CI: 1.08-8.85; *P* = 0.035), LAVI (HR; 1.26 per 10 mL/m^2^ increase; 95% CI: 1.03-1.56; *P* = 0.026) and LARS (HR: 1.09 per 1% decrease; 95% CI: 1.01-1.17; *P* = 0.018) were significantly associated with the composite endpoint (Table 5). The presence of LV obstruction, as well as LA pump strain and LA conduit strain, was not associated with the composite endpoint. As shown in Figure 3, Kaplan-Meier analysis demonstrated significant differences among the groups (log-rank *P* = 0.013). Integrating genotype and LARS identified sarcomere-positive patients with low LARS as a high-risk population compared to the others (HR: 4.76; 95% CI: 1.59-14.2; *P* = 0.005).

**Table 5:** Cox Regression Model for Evaluating Association With Composite of LVSD, Hospitalization for Heart Failure and All-Cause Death

	HR (95% CI)	*P* Value
Age at echocardiography, per 10-y increase	0.99 (0.72-1.36)	0.96
Female sex	0.99 (0.34-2.87)	0.99
Body mass index, per 1 kg/m^2^ increase	1.00 (0.88-1.14)	0.96
Sarcomere positive	3.10 (1.08-8.85)	0.035
LV maximal wall thickness, per 1 mm increase	0.99 (0.87-1.12)	0.83
LV end-diastolic volume, per 10 mL increase	1.03 (0.93-1.15)	0.56
LV ejection fraction, per 1% decrease	1.05 (0.97-1.14)	0.23
LV global longitudinal strain, per 1% increase	1.12 (0.96-1.29)	0.15
Septal E/e’ ratio, per 1 increase	1.01 (0.92-1.11)	0.84
LV obstruction	0.24 (0.03-1.84)	0.17
LA volume index, per 10 mL/m^2^ increase	1.26 (1.03-1.56)	0.026
LA reservoir strain, per 1% decrease	1.09 (1.01-1.17)	0.018
LA pump strain, per 1% decrease	1.12 (1.00-1.25)	0.052
LA conduit strain, per 1% decrease	1.06 (0.96-1.17)	0.25
Follow-up period, per 1 y increase	0.90 (0.73-1.12)	0.36

Abbreviations as in Tables 3 and 4.

## Discussion

In this study, we conducted myocardial strain analysis using speckle-tracking echocardiography in more than 100 genotyped HCM patients. Consistent with prior studies, our HCM cohort showed that a significant proportion of patients had impaired LVGLS and LARS despite preserved LVEF, highlighting the potential utility of strain parameters in early disease assessment. The main findings are as follows.

1) LVGLS and LARS were not associated with genotype positivity.
2) Lower LARS was associated with the progression to LVSD and adverse clinical outcomes and its integration with genotyping may contribute to risk stratification (Central Illustration).

**Central Illustration:**
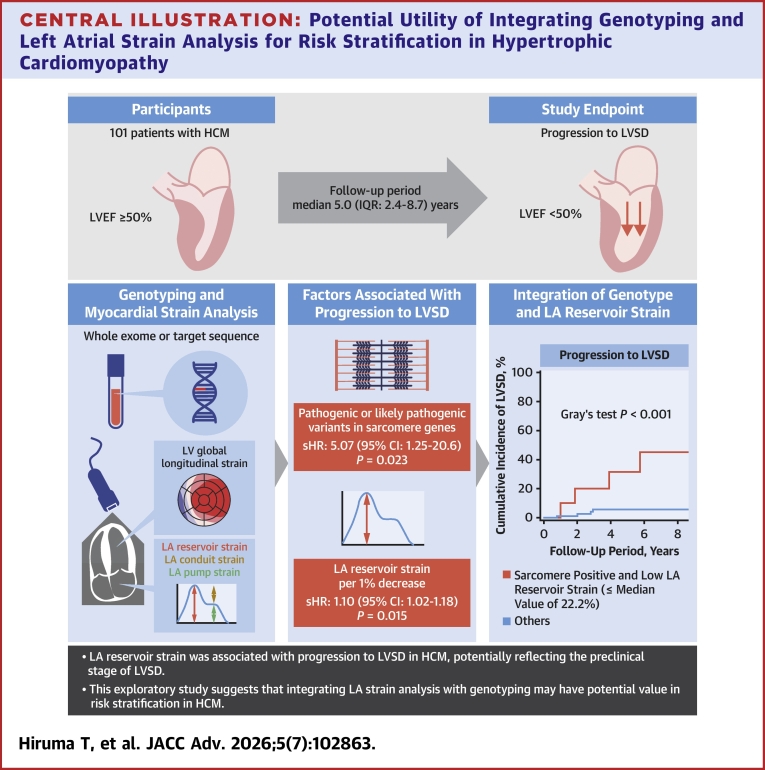
**Potential Utility of Integrating Genotyping and Left Atrial Strain Analysis for Risk Stratification in Hypertrophic Cardiomyopathy** A total of 101 patients with HCM and preserved LVEF were included in this study. All patients underwent genetic testing and myocardial strain analysis. Genotype and LARS, but no LVGLS, were significantly associated with the progression to LVSD. Integrating LARS with genotype may have potential value in risk stratification for the progression to LVSD, potentially reflecting a preclinical stage of LVSD. HCM = hypertrophic cardiomyopathy; LA = left atrium; LVEF = left ventricular ejection fraction; LV = left ventricle; LVSD = left ventricular systolic dysfunction; sHR = subdistribution HR.

### Genotype positivity-strain relationship in HCM

Genotype is a key determinant of phenotype in HCM, with pathogenic sarcomeric variants having significant impact on age of onset, septal morphology, extent of hypertrophy, and clinical outcomes.^5^^,^^6^ Recent studies have deepened our understanding of genotype-phenotype correlations; however, the relationship between genotype positivity and strain parameters remains poorly explored. In this study, we observed no significant difference in strain parameters between genotype-positive and genotype-negative. In general, LVGLS and LARS decline with age due to various age-related factors (eg increased atrial stiffness, progression of myocardial fibrosis).^23^^,^^30^ In our cohort, genotype-positive patients were significantly younger and therefore might be expected to exhibit better strain values appropriate for their age, but that was not the case. Rather than the age-dependent decline in LVGLS and LARS, the decline in strain parameters would be related to the timing of HCM onset by itself. Early-onset HCM induced the decline in strain values even at a younger age at the same levels as those observed at an older age in the late-onset HCM. A plausible explanation for reduced LVGLS lies in the more pronounced LV hypertrophy in sarcomere-positive patients, as hypertrophied segments are known to exhibit marked reductions in longitudinal strain due to the interstitial fibrosis, a histological hallmark of HCM.^17^^,^^31^ LARS is a sensitive marker of LA function, which reflects reservoir function for pulmonary venous return during LV systole and isovolumic relaxation. Previous electrophysiological study revealed that sarcomere-positive patients exhibit more extensive electroanatomical remodeling than genotype-negative patients, despite being of similar age at the time of catheter ablation for paroxysmal AF.^32^ In line with these findings, the present results indicate that sarcomere-positive individuals demonstrated reduced LARS even at a younger age, possibly reflecting early LA remodeling. To elucidate the relationship between genotype positivity and strain parameters, longitudinal studies on each HCM patient, hopefully before and after the HCM onset, are warranted.

### Predictive factors of LVSD in HCM

Pathogenic sarcomeric variants are major contributors to the development of LVSD in HCM. Importantly, the presence of multiple pathogenic variants in sarcomere-encoding genes or coexisting pathogenic variants in other CVD-associated genes has been shown to confer even greater risk and to accelerate LVSD.^3^^,^^6^ Such multilocus genotypes are relatively small subpopulation, accounting for only 5% to 10% of sarcomere-positive patients. Thus, at present, genotype cannot fully explain the heterogeneity in adverse cardiac remodeling and its utility in clinical setting remains limited.

In this context, our study identified an association between lower LARS and the progression to LVSD. LARS captured a distinct dimension of HCM by reflecting LA remodeling, a pathophysiologic process that has not been thoroughly investigated in the progression to LVSD. Moreover, integrating LARS with genotype was associated with differences in event rates not only for LVSD but also for the composite outcome of LVSD, hospitalization for heart failure, and all-cause death. In this regard, LARS may provide additional insight into phenotypic heterogeneity among sarcomere-positive individuals and may have potential value for risk stratification. Although the exact mechanism underlying the predictive value of LARS for future LVSD remains to be elucidated, a key strength of LARS lies in its ability to capture both LA and LV remodeling. A reduction in LARS primarily reflects LA dysfunction, which typically emerges at the earliest phase of adverse cardiac remodeling. LARS has been incorporated into contemporary LV diastolic function assessment frameworks and has demonstrated utility as a surrogate for LV filling pressure.^33^ In addition, LARS serves as a sensitive indicator of impaired longitudinal shortening of the LV base, which may manifest at the preclinical phase of LVSD.^10^^,^^23^^,^^34^ This dual capacity may underlie the strong prognostic performance of LARS for predicting the progression to LVSD in HCM. Future longitudinal studies are warranted to clarify whether LARS primarily reflects upstream atrial remodeling that precedes LVSD, or whether it directly indicates early subclinical LV involvement. Either scenario underscores the potential of LARS as a prognostic tool for early disease monitoring in HCM.

In contrast, LVGLS, despite being a sensitive indicator of impaired LV systolic function in heart-failure populations,^35^^,^^36^ did not show predictive values for the progression to LVSD in our HCM cohort. This discrepancy may stem from the unique mechanism underlying the decline in LVGLS in HCM, as previously discussed; impaired LVGLS does not necessarily indicate a preclinical stage of LVSD but rather reflects severe hypertrophy in HCM. In this context, LARS may provide additional insight into the early identification of individuals at risk of progressing to LVSD than LVGLS during the early stage of the disease.

Patients who have progressed to LVSD are vulnerable to SCD and drug-refractory heart failure and would benefit from timely consideration of implantable defibrillators and advanced heart failure care.^3^^,^^4^ Proactive identification of patients predisposed to LVSD facilitates preventive strategies. In this context, LARS-based approach offers a valuable means for detecting subclinical deterioration before overt LVSD emerges as well as defining treatment eligibility. Preventive medications for LVSD in HCM are still to be explored. However as shown in the pivotal studies for heart failure with preserved ejection fraction, patients with mildly reduced ejection fraction could benefit from angiotensin receptor-neprilysin inhibitors and mineralocorticoid receptor antagonists, which are established as indispensable medications for heart failure with systolic dysfunction.^37^^,^^38^ LARS-guided therapeutic approach has potential to extend the principles of heart failure management into earlier stages of HCM before reaching conventional categories such as reduced LVEF. However, further prospective interventional study of LARS-guided approach is warranted to verify this concept.

Future investigations should clarify the molecular underpinnings of LA dysfunction, elucidate its temporal relationship with sarcomeric variants, and define the longitudinal trajectory of multichamber remodeling in HCM by integrated genetic and imaging approaches. These efforts will bring tailored management that modifies disease course and improve long-term outcomes in HCM.

### Study Limitations

Several limitations of this study merit consideration. First, this was a single-center, retrospective analysis conducted at a tertiary referral hospital, which may limit the generalizability of the findings to broader HCM populations. In addition, as our hospital is a tertiary care center, the cohort may have been relatively younger; however, the overall echocardiographic parameters were generally comparable to those reported in prior study.^16^ Second, the relatively small number of patients who progressed to the endpoints limited the statistical power for extensive subgroup analyses and may have introduced bias and imprecision in hazard estimation, as reflected by the wide CIs. Accordingly, our findings should be considered exploratory. To address these limitations, external validation in larger, multicenter cohorts encompassing a broader spectrum of patients with HCM is warranted. Third, the temporal relationship between LARS decline and LV systolic deterioration was inferred from baseline data and prospective validation in serial imaging studies is needed to confirm causality. Fourth, the lack of certain data previously reported as prognostic factors of LVSD (eg cardiac troponin levels) limited our ability to fully evaluate the incremental prognostic value of LARS beyond existing markers. Future studies incorporating a broader range of conventional risk factors are warranted to more comprehensively assess the clinical utility of LARS in risk stratification. Fifth, the lack of cardiac magnetic resonance imaging data made it difficult to evaluate the relationship between myocardial strain parameters and myocardial fibrosis. Future studies incorporating comprehensive cardiac magnetic resonance imaging assessment may provide further mechanistic insights into this relationship. Lastly, this study did not adopt existing cutoff values of LARS for classification.^33^ Although these cutoff values are now widely used in the assessment of LV diastolic dysfunction in various clinical settings, they were originally derived from non-HCM populations and their applicability to patients with HCM remains uncertain. Given that the primary aim of this study was to evaluate the prognostic value of LARS for predicting LVSD in patients with HCM, we retained a median-based dichotomization in the analysis. Further large-scale studies are warranted to establish optimal cutoff values specific to HCM populations.

## Conclusions

LARS was associated with the progression to LVSD in patients with HCM, potentially reflecting the preclinical stage of overt LVSD, whereas LVGLS was not. Integrating LA strain analysis with genotyping may have potential value in risk stratification, offering prognostic insights for the early detection and management of high-risk individuals. However, these findings are hypothesis-generating and should be interpreted with caution given the small sample size, limited number of events, and the single-center design and require validation in larger multicenter studies.

Perspectives **COMPETENCY IN MEDICAL KNOWLEDGE:** Myocardial strain analysis, particularly LARS, may provide incremental insight into subclinical myocardial remodeling in HCM. Integration of LARS with genotyping may offer further insight into the risk of progression to LVSD and heart failure-related outcomes.

**TRANSLATIONAL OUTLOOK:** Future studies should evaluate whether incorporating LA strain analysis into clinical workflows can improve early identification of high-risk patients with HCM who develop adverse cardiac remodeling and guide therapeutic decision-making.

## Funding support and author disclosures

This work was supported by grants from 10.13039/501100008667SENSHIN Medical Research Foundation (to Dr Ko, Dr Katoh and Dr Nomura), 10.13039/100008695Japan Foundation for Applied Enzymology (to Dr Dai, Dr Ko, and Dr Nomura), MSD Life Science Foundation (to Dr Ko and Dr Nomura), Sakakibara Heart Foundation Cardiovascular Research Program 2023 (to Dr Ko), 10.13039/501100005072Japanese Circulation Society (to Dr Dai, Dr Ko, and Dr Nomura), 10.13039/100007449Takeda Science Foundation (to Dr Ko and Dr Nomura), Cardiovascular Research Fund (to Dr Dai), 10.13039/100020107Hirose Foundation (to Dr Dai), 10.13039/501100005865Mochida Memorial Foundation for Medical and Pharmaceutical Research (to Dr Ko), 10.13039/501100013642Japan Heart Foundation (to Dr Ko and Dr Katoh), 10.13039/501100005927Daiichi Sankyo Foundation of Life Science (to Dr Ko), The Cell Science Research Foundation (to Dr Ko), a Grant-in-Aid for Scientific Research (A) (JP22H00471, JP25H01050) (to Dr Nomura), a Grant-in-Aid for Scientific Research (S) (JP21H05045) (to Dr Komuro), 10.13039/501100001691JSPS KAKENHI Grant Number JP24K23940, (Dr Nomura), JP23KJ0434 and JP25K19380 (to Dr Ko), 24K11211 (Dr Katoh), UTEC-UTokyo FSI Research Grant Program (to Dr Nomura), JST FOREST Program (Grant Number JPMJFR210U) (to Dr Nomura), JSPS Grant-in-Aid for JSPS fellow (to Dr Hiruma and Dr Abe), and 10.13039/100009619Japan Agency for Medical Research and Development (JP18km0405209, JP21ek0109543, JP22ama121016, JP22bm1123011, JP23tm0724607, JP223fa627011, JP22ek0109617, JP23tm0524009, JP23tm0524004, JP23jf0126003, JP24ek0109755, JP24ek0210205, JP24jf0126011, JP25bk0104192, JP25ek0109795, JP26gm4010034) (to Dr Nomura and Dr Komuro), (JP24ek0109777h0001, 25jm0610110h0001, 256f0137004j0001) (to Dr Ko), (JP25ek0109830, JP25bm1123080, JP25ak0101266) (to Dr Katoh). All other authors have reported that they have no relationships relevant to the contents of this paper to disclose.
