# Supplementary material for: Integrating Genotyping and Left Atrial Strain Analysis Enhances Risk Stratification in Hypertrophic Cardiomyopathy
Source: JACC Adv. 2026 Jun 22;5(7):102863. doi: 10.1016/j.jacadv.2026.102863 (PMC13315677; doi:10.1016/j.jacadv.2026.102863)
Supplement: Supplemental_Material [file mmc1.docx]

**Supplementary Appendix**

**Title of the manuscript: Integrating Genotyping and Left Atrial Strain Analysis**

**Enhances Risk Stratification in Hypertrophic Cardiomyopathy**

**1. Supplementary methods**

**2. Tables**

**S1** List of genes analyzed in this study

**S2** Extent of missing data

**S3** Demographic data of the patients with LVSD at the initial evaluation

**S4** Echocardiographic parameters of the patients with LVSD at the initial evaluation

**S5** Detailed data of patients who progressed to LVSD during follow-up period

**S6** Assessment of the proportional subdistribution hazards assumption for Fine–Gray models

**S7** Assessment of the proportional hazards assumption for Cox models

**3. References**

**1. Supplementary methods**

The quality of the purified DNA was assessed using a Qubit Fluorometer (Invitrogen, Carlsbad, CA, USA). Targeted sequencing was performed in 30 patients using a custom panel targeting exons and splicing regions of 83 genes associated with various cardiomyopathies and arrhythmias (**Supplementary Table 1**). Variant annotation was converted from GRCh37/hg19 to GRCh38/hg38 using the liftOver tool from the University of California Santa Cruz Genomics Institute. For the remaining 121 patients, whole-exome sequencing was conducted. DNA libraries were prepared using 50 ng of genomic DNA per sample with the Twist Comprehensive Exome Kit (Twist Bioscience, South San Francisco, CA, USA). Library preparation followed the protocol provided by Twist Bioscience, including enzymatic fragmentation, end repair, ligation with Twist unique dual-index primers, purification and hybridization with the capture probe. Sequencing was performed on a NovaSeq 6000 system (Illumina, San Diego, CA, USA) and read quality was assessed using FastQC software. All raw sequencing data were aligned to the human reference genome (GRCh38) using the Burrows–Wheeler Aligner-MEM algorithm. To detect variants from whole-exome sequencing data, we followed the best practices of the Genome Analysis Toolkit (GATK). Variant calling was performed using GATK HaplotypeCaller with a minimum 20-fold coverage and annotated with ANNOVAR [S1, S2]. Rare variants were filtered by applying a minor allele frequency threshold of <1% from variant databases, including the East Asian population database from the 1000 Genomes Project [S3] and the Tohoku Medical Megabank Organization database [S4]. Subsequently, we extracted variants predicted to alter protein structure or function, including nonsynonymous variants, nonsense variants and splice-site variants, as well as in-frame and frameshift deletions and insertions. The potential impact of variants on splicing was further assessed using SpliceAI [S5] When family members were available, co-segregation analysis was performed to determine whether the variant segregated with the disease phenotype. Finally, variant pathogenicity was evaluated and classified as pathogenic, likely pathogenic, variant of uncertain significance, benign, or likely benign, according to the American College of Medical Genetics and Genomics consensus guidelines and the latest recommendations from ClinGen [24,25].

**2. Tables**

**Table S1. List of genes analyzed in this study.**

| **Condition** | **Genes** |  | |  | |  | |  | |  | |  | |  | |  | |  |
| --- | --- | --- | --- | --- | --- | --- | --- | --- | --- | --- | --- | --- | --- | --- | --- | --- | --- | --- |
| HCM | *MYBPC3* | *MYH7* | | *MYL2* | | *MYL3* | | *TNNI3* | | *TNNT2* | | *TPM1* | | *ACTC1* | | *ACTN2* | | *CSRP3* |
|  | *TNNC1* | *ALPK3* | | *PLN* | | *PRKAG2* | | *ABCC9* | | *CACNA1C* | | *CAV3* | | *CRYAB* | | *FHL1* | | *FXN* |
|  | *GAA* | *GLA* | | *JPH2* | | *LAMP2* | | *LDB3* | | *MYH6* | | *MYOZ2* | | *NEXN* | | *PDLIM3* | | *RAF1* |
|  | *RYR2* | *TCAP* | | *TTR* | | *VCL* | |  | |  | |  | |  | |  | |  |
| DCM | *TTN* | *LMNA* | | *TNNT2* | | *BAG3* | | *FLNC* | | *MYH7* | | *PLN* | | *RBM20* | | *SCN5A* | | *TNNC1* |
|  | *DES* | *DSP* | | *ABCC9* | | *ACTC1* | | *ANKRD1* | | *ACTN2* | | *CRYAB* | | *CSRP3* | | *DMD* | | *EMD* |
|  | *EYA4* | *FKRP* | | *FKTN* | | *ILK* | | *JPH2* | | *JUP* | | *LDB3* | | *LMNA* | | *MIB1* | | *MYBPC3* |
|  | *MYH6* | *MYL2* | | *MYL3* | | *MYPN* | | *NEBL* | | *NEXN* | | *NKX2-5* | | *NPPA* | | *NRAP* | | *PDLIM3* |
|  | *RYR2* | *SDHA* | | *SYNE1* | | *TBX20* | | *TCAP* | | *TNNI3* | | *TPM1* | | *VCL* | |  | |  |
| ACM | *DSC2* | *DSG2* | | *DSP* | | *PKP2* | | *TMEM43* | | *CDH2* | | *JUP* | | *LMNA* | | *MYBPC3* | | *MYH7* |
|  | *MYL3* | *PLN* | | *SCN5A* | | *TGFB3* | | *TTN* | |  | |  | |  | |  | |  |
| NDLVC | *ACTC1* | *ACTN2* | | *DES* | | *DMD* | | *DSP* | | *FLNC* | | *HCN4* | | *ILK* | | *LDB3* | | *LMNA* |
|  | *MIB1* | *MYBPC3* | | *MYH7* | | *MYL2* | | *MYL3* | | *NKX2-5* | | *PLN* | | *RYR2* | | *RBM20* | | *SCN5A* |
|  | *TBX20* | *TMEM43* | | *TNNT2* | | *TPM1* | | *TTN* | |  | |  | |  | |  | |  |
| LQT | *KCNH2* | *KCNQ1* | | *SCN5A* | | *AKAP9* | | *ANK2* | | *CAV3* | | *CACNA1C* | | *KCNE1* | | *KCNE2* | | *KCNJ2* |
| BrS | *SCN5A* | *CACNA1C* | | *CACNB2* | | *HCN4* | | *KCND3* | | *SCN1B* | |  | |  | |  | |  |
| AF | *ABCC9* | *CASQ2* | | *GJA5* | | *KCNA5* | | *KCNQ1* | | *LMNA* | | *NPPA* | | *RYR2* | | *SCN1B* | | *SCN3B* |
|  | *SCN4B* | | *SCN5A* | |  | |  |  |  | |  | |  | |  | |  | |

ACM, arrhythmogenic cardiomyopathy; AF, atrial fibrillation; BrS, Brugada syndrome; DCM, dilated cardiomyopathy; HCM, hypertrophic cardiomyopathy; LQT, long QT syndrome; NDLVC, non-dilated left ventricular cardiomyopathy.

**Table S2. Extent of missing data**

| **Variable** | **Missing number (%)** |
| --- | --- |
| Cardiac troponin I | 32 (31.7) |
| LV global longitudinal strain | 14 (13.9) |
| C-reactive protein | 7 (6.9) |
| Lateral E/e’ ratio | 7 (6.9) |
| Septal E/e’ ratio | 4 (4.0) |
| E wave | 2 (2.0) |
| LA pump strain | 2 (2.0) |
| LA conduit strain | 2 (2.0) |
| Estimated glomerular filtration rate | 1 (1.0) |
| B-type natriuretic peptide | 1 (1.0) |
| Age at diagnosis of HCM | 0 (0.0) |
| Age at echocardiography | 0 (0.0) |
| Sex | 0 (0.0) |
| Body mass index | 0 (0.0) |
| Family history of HCM | 0 (0.0) |
| Family history of SCD | 0 (0.0) |
| NYHA functional class | 0 (0.0) |
| The HCM Risk-SCD score | 0 (0.0) |
| Prior unexpected syncope | 0 (0.0) |
| Prior hospitalization for heart failure | 0 (0.0) |
| History of atrial fibrillation | 0 (0.0) |
| Sarcomere positive | 0 (0.0) |
| Other CVD-related variants positive | 0 (0.0) |
| Hypertension | 0 (0.0) |
| Diabetes | 0 (0.0) |
| Dyslipidemia | 0 (0.0) |
| Chronic kidney disease | 0 (0.0) |
| Coronary artery disease | 0 (0.0) |
| Hemoglobin | 0 (0.0) |
| Beta-blocker | 0 (0.0) |
| ACE inhibitor, ARB, or ARNI | 0 (0.0) |
| Mineralocorticoid receptor antagonist | 0 (0.0) |
| Non-dihydropyridine CCB | 0 (0.0) |
| Type I antiarrhythmic agents | 0 (0.0) |
| Type III antiarrhythmic agents | 0 (0.0) |
| Diuretics | 0 (0.0) |
| Anticoagulant agents | 0 (0.0) |
| Permanent pacemaker | 0 (0.0) |
| Implantable cardioverter-defibrillator | 0 (0.0) |
| Septal morphology | 0 (0.0) |
| Interventricular septal wall thickness | 0 (0.0) |
| Posterior wall thickness | 0 (0.0) |
| LV maximal wall thickness | 0 (0.0) |
| LV end-diastolic volume | 0 (0.0) |
| LV end-systolic volume | 0 (0.0) |
| LV ejection fraction | 0 (0.0) |
| Intra LV pressure gradient | 0 (0.0) |
| Intra LV obstruction | 0 (0.0) |
| Apical hypertrophy | 0 (0.0) |
| Apical aneurysm | 0 (0.0) |
| LA diameter | 0 (0.0) |
| LA volume index | 0 (0.0) |
| LA reservoir strain | 0 (0.0) |
| Aortic regurgitation | 0 (0.0) |
| Mitral regurgitation | 0 (0.0) |
| Tricuspid regurgitation | 0 (0.0) |

ACE, angiotensin-converting enzyme; ARB, angiotensin II receptor blocker; ARNI, angiotensin receptor-neprilysin inhibitor; CCB, calcium-channel blocker; CVD, cardiovascular disease; GFR, glomerular filtration rate; HCM, hypertrophic cardiomyopathy; LA, left atrium; LV, left ventricle; NYHA, New York Heart Association; SCD, sudden cardiac death.

**Table S3. Demographic data of the patients with LVSD at the initial evaluation**

| **Variable** | **N** | **Patients with LVSD**  **(N = 29)** |
| --- | --- | --- |
| Age at diagnosis of HCM, years | 29 | 35 (16-60) |
| Age at echocardiography, years | 29 | 54 (40-62) |
| Female sex, % | 29 | 8 (27.6) |
| Body mass index, kg/m^2^ | 29 | 22.2 (19.5-24.5) |
| Family history of HCM, % | 29 | 8 (27.6) |
| Family history of SCD, % | 29 | 4 (13.8) |
| NYHA function class Ⅲ/IV, % | 29 | 25 (86.2) |
| The HCM Risk-SCD score, % | 29 | 3.96 (3.32-5.44) |
| Prior unexpected syncope, % | 29 | 5 (17.2) |
| Prior hospitalization for heart failure, % | 29 | 14 (48.3) |
| History of atrial fibrillation | 29 |  |
| Paroxysmal, % |  | 9 (31.0) |
| Persistent, % |  | 6 (20.7) |
| **Genotype** |  |  |
| Sarcomere positive, % | 29 | 18 (62.1) |
| *MYH7*, % |  | 7 (24.1) |
| *MYBPC3*, % |  | 4 (13.8) |
| *TNNT2*, % |  | 3 (10.3) |
| *MYL2*, % |  | 1 (3.4) |
| *MYL3*, % |  | 1 (3.4) |
| Multiple pathogenic/likely pathogenic variants, % |  | 2 (6.9) |
| Other CVD-related variants positive, % | 29 | 1 (3.4) |
| **Comorbidities** |  |  |
| Hypertension, % | 29 | 9 (31.0) |
| Diabetes, % | 29 | 6 (20.7) |
| Dyslipidemia, % | 29 | 11 (37.9) |
| Chronic kidney disease, % | 29 | 1 (3.4) |
| Coronary artery disease, % | 29 | 5 (17.2) |
| **Blood test at echocardiography** |  |  |
| Hemoglobin, g/dL | 29 | 13.6 (11.9-15.1) |
| Estimated GFR, mL/min/1.73m^2^ | 29 | 62.7 (40.4-81.0) |
| C-reactive protein, mg/dL | 29 | 0.07 (0.04-0.18) |
| B-type natriuretic peptide, pg/mL | 29 | 420 (230-765) |
| Cardiac troponin I, pg/mL | 10 | 88.4 (36.1-176.2) |
| **Medications at echocardiography** |  |  |
| Beta-blocker, % | 29 | 26 (89.7) |
| ACE inhibitor, ARB or ARNI, % | 29 | 22 (75.9) |
| Mineralocorticoid receptor antagonist, % | 29 | 15 (51.7) |
| Diuretics, % | 29 | 1 (3.4) |
| Non-dihydropyridine CCB, % | 29 | 1 (3.4) |
| Type I antiarrhythmic agents, % | 29 | 0 (0.0) |
| Type III antiarrhythmic agents, % | 29 | 9 (31.0) |
| Anticoagulant agents, % | 29 | 15 (51.7) |
| **Cardiac electronic implanted devices at echocardiography** | | |
| Permanent pacemaker, % | 29 | 2 (6.9) |
| Implantable cardioverter-defibrillator, % | 29 | 6 (20.7) |

Data are expressed as median (interquartile range) or number of patients with percentage.

ACE, angiotensin-converting enzyme; ARB, angiotensin II receptor blocker; ARNI, angiotensin receptor-neprilysin inhibitor; CCB, calcium-channel blocker; CVD, cardiovascular disease; GFR, glomerular filtration rate; HCM, hypertrophic cardiomyopathy; LVSD, left ventricular systolic dysfunction; NYHA, New York Heart Association; SCD, sudden cardiac death.

**Table S4. Echocardiographic parameters of the patients with LVSD at the initial evaluation**

| **Variable** | **N** | **Patients with LVSD** |
| --- | --- | --- |
| **Septal morphology** | 29 |  |
| Reverse curve septum, % |  | 9 (31.0) |
| Sigmoidal septum, % |  | 2 (6.9) |
| Apical dominant, % |  | 1 (3.4) |
| Neutral, % |  | 17 (58.6) |
| **LV parameters** |  |  |
| Interventricular septal wall thickness, mm | 29 | 11 (9-14) |
| Posterior wall thickness, mm | 29 | 9 (7-11) |
| LV maximal wall thickness, mm | 29 | 15 (14-16) |
| LV end-diastolic volume, mL | 29 | 143 (96.1-178) |
| LV end-systolic volume, mL | 29 | 87.0 (56.0-117) |
| LV ejection fraction, % | 29 | 37 (31-44) |
| LV global longitudinal strain, % | 28 | -9.9 (-11.7/-8.0) |
| E wave, cm/sec | 28 | 47.6 (31.2-71.2) |
| Septal E/e’ ratio | 27 | 14.7 (8.6-19.1) |
| Lateral E/e’ ratio | 25 | 10.3 (7.1-13.9) |
| Intra-LV obstruction, % | 29 | 0 (0.0) |
| Apical hypertrophy, % | 29 | 0 (0.0) |
| **LA parameters** |  |  |
| LA diameter, mm | 29 | 49.0 (46.0-54.0) |
| LA volume index, mL/m^2^ | 29 | 72.8 (44.5-79.8) |
| LA reservoir strain, % | 29 | 9.2 (6.0-14.2) |
| LA pump strain, % | 27 | 3.6 (1.8-9.0) |
| LA conduit strain, % | 27 | 4.9 (4.0-7.4) |
| **Valvular heart disease** |  |  |
| Aortic regurgitation ≥ grade 2+, % | 29 | 1 (3.4) |
| Mitral regurgitation ≥ grade 2+, % | 29 | 5 (17.2) |
| Tricuspid regurgitation ≥ grade 2+ % | 29 | 7 (24.1) |

Data are expressed as median (interquartile range) or number of patients with percentage.

HCM, hypertrophic cardiomyopathy; LA, left atrium; LV, left ventricle.

**Tables S5. Detailed data of patients who progressed to LVSD during follow-up period.**

| **Patient** | **Sex** | **Age at**  **echo** | **FH**  **HCM** | **Type of**  **baseline AF** | **Genotype** | **Septal morphology** | **LVMWT**  **(mm)** | **LVEDV**  **(mL)** | **LVEF**  **(%)** | **LAVI**  **(mL/m^2^)** | **LVGLS**  **(%)** | **LARS**  **(%)** |
| --- | --- | --- | --- | --- | --- | --- | --- | --- | --- | --- | --- | --- |
| 1 | F | 52 | − | paroxysmal | *MYBPC3* | reverse curve | 23 | 150 | 67.8 | 76.1 | -16.4 | 12.8 |
| 2 | F | 29 | − | paroxysmal | *TNNI3* | reverse curve | 14 | 124 | 59.1 | 71.1 | -16.7 | 16.4 |
| 3 | M | 67 | − | paroxysmal | *−* | apical | 11 | 101 | 57.4 | 39.6 | -20.7 | 21.3 |
| 4 | M | 78 | − | − | *−* | neutral | 14 | 108 | 66.8 | 44.0 | -12.5 | 26.5 |
| 5 | M | 71 | − | persistent | *−* | reverse curve | 21 | 66 | 59.1 | 31.3 | -12.5 | 8.2 |
| 6 | F | 40 | + | − | *MYH7* | reverse curve | 12 | 78 | 50.0 | 67.1 | -17.6 | 11.1 |
| 7 | M | 56 | − | − | *MYBPC3* | reverse curve | 12 | 130 | 55.4 | 56.2 | -15.0 | 14.0 |
| 8 | F | 43 | + | − | *TNNT2* | neutral | 16 | 73 | 65.8 | 40.6 | -14.9 | 22.4 |

AF, atrial fibrillation; F, female; FH, family history; HCM, hypertrophic cardiomyopathy; LARS, left atrial reservoir strain; LAVI, left atrial volume index; LVEDV, left ventricular end-diastolic volume; LVEF, left ventricular ejection fraction; LVGLS, left ventricular global longitudinal strain; LVMWT, left ventricular maximal wall thickness; LVSD, left ventricular systolic dysfunction; M, male.

**S6. Assessment of the proportional subdistribution hazards assumption for Fine–Gray models**

| **Variable** | **Number of tested** | **Median**  ***p*-value** | **Number of tests with *p* < 0.05** |
| --- | --- | --- | --- |
| Sarcomere positive | 20 | 0.412 | 0 |
| Age at diagnosis of HCM | 20 | 0.086 | 0 |
| Age at echocardiography | 20 | 0.091 | 0 |
| Female sex | 20 | 0.025 | 20 |
| Body mass index | 20 | 0.504 | 0 |
| Family history of HCM | 20 | 0.196 | 0 |
| Family history of SCD | 20 | 0.854 | 0 |
| History of atrial fibrillation | 20 | <0.001 | 20 |
| Hypertension | 20 | <0.001 | 20 |
| Diabetes | 20 | 0.775 | 0 |
| Dyslipidemia | 20 | 0.455 | 0 |
| Coronary artery disease | 20 | 0.953 | 0 |
| Chronic kidney disease | 20 | 0.542 | 0 |
| NYHA function class III/IV | 20 | 0.964 | 0 |
| Prior hospitalization for heart failure | 20 | 0.205 | 0 |
| Prior unexpected syncope | 20 | 0.190 | 0 |
| Beta-blocker | 20 | <0.001 | 20 |
| ACE inhibitor, ARB or ARNI | 20 | 0.581 | 0 |
| Mineralocorticoid receptor antagonist | 20 | 0.479 | 0 |
| Non-dihydropyridine CCB | 20 | 0.946 | 0 |
| Type I antiarrhythmic agents | 20 | 0.986 | 0 |
| Type III antiarrhythmic agents | 20 | <0.001 | 20 |
| Diuretics | 20 | 0.005 | 20 |
| Anticoagulant agents | 20 | 0.231 | 0 |
| Hemoglobin | 20 | 0.180 | 0 |
| Estimated GFR | 20 | 0.118 | 0 |
| C-reactive protein | 20 | 0.122 | 4 |
| B-type natriuretic peptide | 20 | 0.499 | 0 |
| Cardiac troponin I | 20 | 0.260 | 5 |
| Permanent pacemaker | 20 | 0.969 | 0 |
| Implantable cardioverter-defibrillator | 20 | 0.293 | 0 |
| Other CVD-related variants positive | 20 | <0.001 | 20 |
| HCM Risk-SCD score | 20 | 0.171 | 0 |
| Interventricular septal thickness | 20 | 0.476 | 0 |
| Posterior wall thickness | 20 | 0.098 | 0 |
| LV maximal wall thickness | 20 | 0.142 | 0 |
| LV end-diastolic volume | 20 | 0.381 | 0 |
| LV end-systolic volume | 20 | 0.544 | 0 |
| LV ejection fraction | 20 | 0.761 | 0 |
| E wave | 20 | 0.800 | 0 |
| Septal E/e’ ratio | 20 | 0.495 | 0 |
| Lateral E/e’ ratio | 20 | 0.144 | 0 |
| LA diameter | 20 | 0.279 | 0 |
| LA volume index | 20 | 0.059 | 0 |
| Intra-LV obstruction | 20 | 0.885 | 0 |
| Intra-LV pressure gradient | 20 | 0.082 | 0 |
| Apical hypertrophy | 20 | 0.987 | 0 |
| Mitral regurgitation ≥ grade 2+ | 20 | 0.973 | 0 |
| Tricuspid regurgitation ≥ grade 2+ | 20 | 0.998 | 0 |
| Apical aneurysm | 20 | <0.001 | 20 |
| LV global longitudinal strain | 20 | 0.062 | 2 |
| LA reservoir strain | 20 | 0.376 | 0 |
| LA pump strain | 20 | 0.402 | 0 |
| LA conduit strain | 20 | 0.888 | 0 |

ACE, angiotensin-converting enzyme; ARB, angiotensin II receptor blocker; ARNI, angiotensin receptor-neprilysin inhibitor; CCB, calcium-channel blocker; CVD, cardiovascular disease; GFR, glomerular filtration rate; HCM, hypertrophic cardiomyopathy; LA, left atrium; LV, left ventricle; NYHA, New York Heart Association; SCD, sudden cardiac death.

**S7. Assessment of the proportional hazards assumption for Cox models**

| **Variable** | **Number of tested** | **Median**  ***p*-value** | **Number of tests with *p* < 0.05** |
| --- | --- | --- | --- |
| Sarcomere positive | 20 | 0.132 | 0 |
| Age at diagnosis of HCM | 20 | 0.244 | 0 |
| Age at echocardiography | 20 | 0.307 | 0 |
| Female sex | 20 | 0.597 | 0 |
| Body mass index | 20 | 0.543 | 0 |
| Family history of HCM | 20 | 0.997 | 0 |
| Family history of SCD | 20 | 0.491 | 0 |
| History of atrial fibrillation | 20 | 0.001 | 20 |
| Hypertension | 20 | 0.612 | 0 |
| Diabetes | 20 | 0.169 | 0 |
| Dyslipidemia | 20 | 0.840 | 0 |
| Coronary artery disease | 20 | 1.000 | 0 |
| Chronic kidney disease | 20 | 0.823 | 0 |
| NYHA function class III/IV | 20 | 0.165 | 0 |
| Prior hospitalization for heart failure | 20 | 0.358 | 0 |
| Prior unexpected syncope | 20 | 0.016 | 20 |
| Beta-blocker | 20 | 0.120 | 0 |
| ACE inhibitor, ARB or ARNI | 20 | 0.556 | 0 |
| Mineralocorticoid receptor antagonist | 20 | 0.138 | 0 |
| Non-dihydropyridine CCB | 20 | 0.098 | 0 |
| Type I antiarrhythmic agents | 20 | 1.000 | 0 |
| Type III antiarrhythmic agents | 20 | 0.030 | 20 |
| Diuretics | 20 | 0.245 | 0 |
| Anticoagulant agents | 20 | 0.388 | 0 |
| Hemoglobin | 20 | 0.051 | 0 |
| Estimated GFR | 20 | 0.787 | 0 |
| C-reactive protein | 20 | 0.262 | 0 |
| B-type natriuretic peptide | 20 | 0.404 | 0 |
| Cardiac troponin I | 20 | 0.622 | 0 |
| Permanent pacemaker | 20 | 0.346 | 0 |
| Implantable cardioverter-defibrillator | 20 | 0.053 | 0 |
| Other CVD-related variants positive | 20 | 0.016 | 20 |
| HCM Risk-SCD score | 20 | 0.084 | 0 |
| Interventricular septal thickness | 20 | 0.885 | 0 |
| Posterior wall thickness | 20 | 0.211 | 0 |
| LV maximal wall thickness | 20 | 0.139 | 0 |
| LV end-diastolic volume | 20 | 0.447 | 0 |
| LV end-systolic volume | 20 | 0.613 | 0 |
| LV ejection fraction | 20 | 0.613 | 0 |
| E wave | 20 | 0.086 | 0 |
| Septal E/e’ ratio | 20 | 0.594 | 0 |
| Lateral E/e’ ratio | 20 | 0.046 | 11 |
| LA diameter | 20 | 0.627 | 0 |
| LA volume index | 20 | 0.238 | 0 |
| Intra-LV obstruction | 20 | 0.960 | 0 |
| Intra-LV pressure gradient | 20 | 0.567 | 0 |
| Apical hypertrophy | 20 | 0.880 | 0 |
| Mitral regurgitation ≥ grade 2+ | 20 | 1.000 | 0 |
| Tricuspid regurgitation ≥ grade 2+ | 20 | 1.000 | 0 |
| Apical aneurysm | 20 | 0.635 | 0 |
| LV global longitudinal strain | 20 | 0.841 | 0 |
| LA reservoir strain | 20 | 0.949 | 0 |
| LA pump strain | 20 | 0.061 | 0 |
| LA conduit strain | 20 | 0.070 | 0 |

Abbreviations are as defined in **Supplementary Table 6**.

**3. References**

S1 Poplin R, Ruano-Rubio V, DePristo MA, et al. Scaling accurate genetic variant discovery to tens of thousands of samples [preprint]. bioRxiv 2017.

S2 Wang K, Li M, Hakonarson HH. ANNOVAR: Functional annotation of genetic variants from high-throughput sequencing data. Nucleic Acids Res 2010;38:e164.

S3 1000 Genomes Project Consortium, Auton A, Brooks LD, et al. A global reference for human genetic variation. Nature 2015;526:68-74.

S4 Nagasaki M, Yasuda J, Katsuoka F, et al. M. Rare variant discovery by deep whole-genome sequencing of 1,070 Japanese individuals. Nat Commun 2015;6:8018.

S5 Jaganathan K, Kyriazopoulou Panagiotopoulou S, McRae JF, et al. Predicting splicing from primary sequence with deep learning. Cell 2019;176:535-548.e24.
